# Glanders1Read before the Students’ Society of the McKillip Veterinary College.

**Published:** 1901-03

**Authors:** C. P. Liegerot


### GLANDERS.[1]

By C. P. Liegerot.

*Definition.* A contagious disease of the horse, often transmitted to man, asses, mules, sheep, dogs, and most all of the wild animals.

*History.* Glanders (glanders and farcy) is one of the oldest known diseases of horses. Aristotle, Vegetius, and Hippocrates were acquainted with farcy and glanders of the donkey. The Roman author, Apsyrtus, has described glanders, and Vegetius recognized several kinds of glanders (*malleus*) especially that of the nose and skin (*malleus humidus et farciminosus*).

In the middle ages glanders was considered by the German laws to be a legal unsoundness (breach of warranty). The infectiousness of glanders was recognized as early as the seventeenth century. Solleysel (in 1664) supposed it could be transmitted by the air. Van Helmont (1682), a man of great judgment for his day, considered it identical with human syphilis, on account of it breaking out in ulcers over the body and mucous membranes. Even in 1734 Gaspard Saunier gave precise directions about disinfecting stables. Garsault (1741) and Bourgelat (1764) recommended immediate slaughter of horses suffering from glanders and segregation of suspected animals.

The identity of glanders and farcy was clearly recognized at that time. At the end of the eighteenth century Abildgaard and Viborg, in Denmark, proved by a great number of experiments that glanders could be transmitted from one horse to another. These investigators demonstrated at that early period that the virus of glanders was fixed; that it could be transmitted by the respiratory air; that the transfusion of the blood of horses suffering from glanders is far less efficacious for transmitting the disease than inoculations of secretions or of pus, and that the virus loses its power of infection if dried or heated to 45° C. Similar experiments were made in England by Coleman and Delabin Blaine, who recognized that the nasal discharge was the principal vehicle of the virus.

At the beginning of the nineteenth century the contagiousness of glanders was much doubted. Especially in France, the view taken by the Alfort Veterinary College that glanders might arise spontaneously from an attack of strangles was far more widely accepted than the doctrine of its contagiousness, which was stoutly supported by the Veterinary College of Lyons; the consequence

[1] Read before the Students' Society of the McKillip Veterinary College.

being that precautionary regulations against glanders were abolished, with the result that the spread of the disease increased to an extraordinary extent in France. In Williams' *Principles and Practice of Veterinary Medicine* to-day we find the statement that glanders and its variety, farcy, originate spontaneously in the horse, ass, and mule. At one time glanders was looked upon as tuberculosis (Dupuy), or as simple pyæmia (Vatel and Bouley). It was only when Rayer, in 1837, had proved the transmissibility of glanders to man, and when Chauveau, in 1868, had shown that the virus was contained chiefly in the firm, component parts of the infective material, that the fact of the contagiousness of the disease was again accepted.

The theory of spontaneous origin of glanders was accepted in Germany by almost every practitioner at the middle of the nineteenth century. It was believed that glanders could be produced by the injections of pus, and that strangles could turn to glanders. (Herring and Funke). Glanders was looked upon respectively as a tubercular disease, scrofula (Hauboxer), pyæmia, diphtheritis, general dyscrasia, and cachexia. Virchow was the first to declare that the nodules of glanders were independent anatomical formations, which he placed under the head of granulating tumors. Gerlach was the great advocate for the exclusively contagious origin of glanders. He threw much light on the clinical and diagnostic aspect of the disease. We are indebted to Leisering for the first exact anatomical description of glanders.

The first bacteriological researches were made in 1868 by Zura and Harrier, who found a fungus which they believed to be the cause of glanders and to be identical with that of human syphilis. In the same year Christol and Kiener had discovered the microorganism of glanders. Bauchard, Captiain, and Charrin, in 1881 and 1882, sought to isolate the virus of glanders.[1]

*Bacteriology.* The bacillus matter which is the cause of glanders was discovered by Löffler and Schutz, German bacteriologists, in 1882. These scientists obtained the organism in pure culture and proved by experimental inoculation that it is the cause of the disease. Many attempts had been made prior to that time to discover the cause of this disease, but without success. The bacillus of glanders is in the form of straight or slightly curved rods, with rounded ends, and of the same length as the tubercle bacillus, but somewhat thicker. The size, however, is not uniform. Rarely

[1] These investigators found the bacillus of glanders and have equal claim to originality with Löffler and Schutz.—Ed.

filamentous forms occur. The bacillus stains readily with the basic aniline dyes, but the addition of a mordant, as carbolic acid or potassium hydrate, facilitates the staining. It is easily decolorized. It does not stain by Mann's method, being decolorized by the iodine solution. It docs not stain regularly, but shows portions more deeply stained than others. The parts which do not take the stain deeply are frequently of an oval shape, giving an appearance of spores. However, it is not generally supposed that the glanders bacillus produces spores, for the low powers of resistance of the organism are not in accordance with the existence of spores.

The bacillus mallei grows quite readily on most of the ordinary culture media, but gives its characteristic growth on potato. On this medium it shows on the third day a growth closely resembling honey. Subsequently it gradually grows darker until at the eighth day it has a reddish-brown or chocolate color. In bouillon a cloudiness is produced during the first few days, but later the culture sinks to the bottom of the tube in the form of a string deposit. The organism grows at a temperature anywhere between 21° C. and 42° C., but multiplies most rapidly at 35° to 37° C. It is non-motile and grows in the presence of air.

The bacillus of glanders has only moderate powers of resistance to destructive agencies. It withstands putrefaction and drying for two to three weeks. It lives in culture about a month at incubator-temperature, and about four months at room-temperature. Heating for ten minutes to 55° C. or exposure to a 5 per cent. solution of carbolic acid for three minutes will devitalize the organism. Hence boiling momentarily, or the ordinary application of disinfecting solutions, will be sufficient for purposes of disinfection.

In the animal tissues it occurs scattered among the cells and within them, chiefly, however, in the former position. They are quite plentiful in recent lesions, but difficult to find in those of long standing. In advanced cases the bacillus occurs in the blood.

The germs occur in the nasal secretion as well as in the secretion from glanderous ulcers of the skin, but they are here mixed with so many other varieties of bacilli of similar morphology that staining of these products does not afford a reliable means of diagnosis.

Cultures of the bacillus may be obtained by inoculating the suitable media from fresh, uncontaminated lesions, and placing these cultures in the incubator. A method which is most reliable is, first, to inoculate an experimental animal, as a guinea-pig or a field mouse, from the suspected lesions, and then inoculate the culture media from the tissues of this animal.

In nature the bacillus of glanders may occur on any object upon which it has been deposited from the secretions or diseased tissues of an animal or man suffering from glanders, be it bucket, watering-trough or feed-trough, harness, halter, yoke, wagon-pole, fence, litter, food-stuff, or other object. It may exist on these places of deposit for a considerable length of time, perhaps not over four months, if disinfection is not practised.

*Pathogenesis.* The infecting agent is some object which has been contaminated with glanders bacilli, or it may be the diseased animal itself in case it comes into immediate contact with a susceptible animal. The injection may enter the body through the respiratory tract, the skin or mucous membranes, or the digestive tract. The latter mode of entrance is rather rare. When the respiratory tract is the portal of entrance, the germs probably come into contact with it in an admixture with dust. Whether or not the glanders bacilli can penetrate the unabraded mucous membrane is a debatable question, but in all probability they can. The germs may lodge and set up the primary pathological changes at any part of the respiratory tract.

When the skin is the gateway of entrance of the infection it is most likely that a pre-existing abrasion of the skin is necessary in order that the germ may gain a foothold. It is hardly possible that the organism can penetrate the uninjured skin.

It is not considered that the gastric juice is fatal to the glanders bacillus, but experimental feeding with glanderous material has in many cases given negative results. This leads to the impression that infection by the digestive tract does not often occur.

*Pathological Anatomy.* There are two varieties of glanders, depending upon the location, viz., farcy, when located on the skin and subcutaneous lymphatic glands; and glanders proper, when it is of the internal form. The term farcy is one of convenience only, and ought to be abolished entirely.

There are also two forms depending upon the course, viz., acute and chronic.

Chronic glanders is confined principally to the respiratory mucous membrane, lungs, lymphatic glands, skin, and subcutaneous tissue. In the respiratory organs it may be in the form of circumscribed nodules, or it may be diffuse. The nasal mucous membrane is most commonly involved. Here it occurs chiefly in the circumscribed form. The first appearance is that of small, gelatinous, translucent nodules, projecting visibly above the surface, and surrounded by a congested area. They soon undergo a softening process and break down in their centre into a puriform liquid, thus producing an ulcer. These ulcers have ragged, undetermined, indurated edges. Several ulcers may become confluent, forming a large ulcerated surface. The bony wall of the nasal fossa or the septum nasi may become perforated as a result of the ulcerative process. Ulcers may heal. If so, a puckered scar is seen. Ulcers may be confined to the upper parts of the nasal fossæ or to the wall of the false nostril. The lesions may also be found in the sinus of the head, the larynx, trachea, and bronchi. In the diffuse form the lesions take on the character of a catarrhal inflammation with thickening and superficial ulceration of the mucous membrane. In the lungs the disease may occur in either the nodular or the diffuse form. The nodules are translucent, pearl-gray in color, and surrounded by a hyperæmic zone. They may become calcareous or encapsulated. It is said that the lungs, when they are filled with these nodules, feel somewhat like a bag of shot or peas. In the diffuse form large foci several inches in diameter may occur. The lymphatic glands of the thorax become swollen, hardened, and studded with small nodules. In the skin form of the disease the nodules are found in the skin, subcutis, and the intermuscular tissues. These nodules later break down and ulcerate as they do in the respiratory mucous membrane. The superficial lymphatic glands become enlarged and congested and, later on, hardened. This, together with the enlarged lymphatic channels connecting them, gives the appearance of a string of beads. The lymphatic glands in the submaxillary region at first become swollen, but later become contracted, hardened, and attached to the bone beneath and the skin above them. Nodules may also appear in most of the other organs and tissues of the body.

In the acute form of glanders the alterations take the form of rapidly progressing infiltration, ulceration, and gangrene in the various seats of the disease.

